# Making pre-screening for Alzheimer's disease (AD) and Postoperative delirium among post-acute COVID-19 syndrome - (PACS) a national priority: The Deep Neuro Study

**DOI:** 10.12688/openreseurope.15005.1

**Published:** 2022-08-22

**Authors:** Ioannis Tarnanas, Magda Tsolaki

**Affiliations:** 1 Altoida Inc, Washington DC, 20003, USA; 2 Global Brain Health Institute, Trinity College Dublin, Dublin, Ireland; 3 Atlantic Fellow for Equity in Brain Health, University of California San Francisco, San Francisco, USA; 4 Latin American Brain Health Institute (BrainLat), Universidad Adolfo Ibáñez, Santiago de Chile, Chile; 5 Greek Association of Alzheimer's Disease and Related Disorders (GAADRD), Thessaloniki, Greece; 6 1st University Department of Neurology UH “AHEPA”, School of Medicine, Faculty of Health Sciences, Aristotle University of Thessaloniki, Thessaloniki, Greece; 7 Laboratory of Neurodegenerative Diseases, Center for Interdisciplinary Research and Innovation (CIRI - AUTh) Balkan Center, Aristotle University of Thessaloniki, Buildings A & B, Thessaloniki, Greece

**Keywords:** prediagnostic Alzheimer's disease (AD), digital neuro signatures (DNS), prodromal Alzheimer's disease (AD), postoperative delirium, post-acute COVID-19 syndrome (PASC)

## Abstract

SARS-CoV-2 effects on cognition is a vibrant area of active research. Many researchers suggest that COVID-19 patients with severe symptoms leading to hospitalization, sustain significant neurodegenerative injury, such as encephalopathy and poor discharge disposition. However, despite some post-acute COVID-19 syndrome (PACS) case series that have described elevated neurodegenerative biomarkers, no studies have been identified that directly compared levels to those in mild cognitive impairment, non-PACS postoperative delirium patients after major non-emergent surgery or preclinical Alzheimer’s Disease (AD) patients, that have clinical evidence of Alzheimer's without symptoms. According to recent estimates, there may be 416 million people globally on the AD continuum, which include approximately 315 million people with preclinical AD. In light of all the above, a more effective application of digital biomarker and explainable artificial intelligence methodologies that explored amyloid beta, neuronal, axonal, and glial markers in relation to neurological complications in-hospital or later outcomes could significantly assist progress in the field. Easy and scalable subjects’ risk stratification is of utmost importance, yet current international collaboration initiatives are still challenging due to the limited explainability and accuracy to identify individuals at risk or in the earliest stages that might be candidates for future clinical trials. In this open letter, we propose the administration of selected digital biomarkers previously discovered and validated in other EU funded studies to become a routine assessment for non-PACS preoperative cognitive impairment, PACS neurological complications in-hospital or later PACS and non-PACS improvement in cognition after surgery. The open letter also includes an economic analysis of the implications for such national level initiatives. Similar collaboration initiatives could have existing prediagnostic detection and progression prediction solutions pre-screen the stage before and around diagnosis, enabling new disease manifestation mapping and pushing the field into unchartered territory.

## Disclaimer

The views expressed in this article are those of the author(s). Publication in Open Research Europe does not imply endorsement of the European Commission. 

## Introduction

More than 533 million people globally ^1^ are affected by the progression of coronavirus disease 2019 (COVID-19) and still at the time of this writing studies about long-term persistent impacts have been steadily increasing ^2^. Several studies have reported 31%–69% ^3^ of all COVID-19 patients having experiences post-acute COVID-19 syndrome or PACS with diverse manifestations demonstrating a variety of neurological and neuropsychiatric complications, often referred to as “brain fog” ^4^. Among those complications are chronic fatigue, anosmia, ageusia, dysautonomia and a general cognitive deterioration with or without fluctuations, which could manifest as difficulties with concentration, memory, receptive language and/or executive function ^5– 7^. Interestingly, younger people are also at risk for brain fog, even in the absence of serious disease ^8^. To make things worse, a recent global estimation on the newly estimated total number of people across the Alzheimer's disease continuum, predicts that there may be 416 million people globally on the Alzheimer’s disease continuum, which includes approximately 315 million people with preclinical Alzheimer’s disease (no obvious signs or symptoms, but clinical evidence of Alzheimer's), 69 million people with prodromal (experiencing mild cognitive impairment) and 32 million people meeting the criteria for a clinical diagnosis of dementia or probable/possible Alzheimer’s disease dementia ^9^. Taken together 22% of all persons aged 50 and above or around 416 million people globally exist across the AD continuum. Considering both PACS and the preclinical Alzheimer’s disease population above, we are faced with an unprecedented almost one billion people potentially at risk around their brain health.

### PACS long-term cognitive outcomes

Some recent studies have linked the inflammatory response during PACS with tau hyperphosphorylation leading to neuropathological pathways blockages typically associated with AD ^10^. The clinical phenotype associated with those blockages is a persistent brain fog, often correlated with the severity of PACS symptoms during the acute illness phase ^11, 12^. For example, in about 74% of patients that have been hospitalized due to COVID-19, we observe coordination deficits, that could be explained by tau pathology in the cerebellum ^13^. Additionally, the constellation of long-lasting cognitive symptoms has been linked with leaky ryanodine receptor 2 (RyR2) even following mild illness and across all ages10. A recent comprehensive investigation ^14^ in the context of brain fog within patients that died from COVID-19, has identified extensive brain degeneration and inflammation confirming findings of the previous study, in terms of increased tau pathophysiology10. Consistent with this evidence, a potential molecular mechanism was proposed to explain long-lasting brain fog, which implicates leaking RyR2 activity, defective intracellular Ca2+ regulation caused by mitochondria overload, leading to dysfunction and oxidative stress contributing to AD-like neuropathology ^15, 16^. The link between tau phosphorylation and brain fog observed in these patients is occurring in patients independently of age and brain areas in the cerebellum, which usually doesn’t exhibit tau pathology in typical AD patients. The increased tau signaling alongside TGF-β with their molecular signatures in this context is associated with long-lasting cognitive symptoms (>6 mo), and needs further exploration. A related question is whether the neurological symptoms observed after PACS can be progressive (>6 mo), possibly increasing AD or cognitive impairment risk in the future.

In contrast, biochemical features of resilient cognition, including cellular and synaptic networks in AD have been positively associated with cognition, representing a "signature" of brain resilience ^17, 18^. Data presented from the Mayo Clinic Study of Aging (MCSA), suggests that there are molecular signatures such as FDG-PET uptake in the bilateral anterior cingulate cortex and anterior temporal pole, which has been associated with stable global cognition in individuals 80+. This molecular signature, named by the authors the resilience signature, provided significant information about global longitudinal cognitive change trajectories when examining both the MCSA and ADNI cohorts, even accounting for the amyloid status ^19^. Such findings and some preventive factors, in particular of the control of systemic vascular risk should be taken into consideration when trying to isolate PACS-anticipating risk factors at the time of initial COVID-19 diagnosis. Currently the four known factors are Epstein-Barr virus viremia, SARS-CoV-2 RNAemia, type 2 diabetes, and specific auto-antibodies ^20^. For example, dysregulated glucose metabolism during the initial COVID-19 diagnosis may be inducing cognitive disruption in both COVID-19 and AD and could be considered a common diagnostic biomarker. Taken together, the observed biochemical changes associated with long or short-term brain fog should be assessed and regularly monitored. Moreover, several genetic polymorphisms associated with increased cognitive impairment risk are currently lacking and a careful investigation within each patient's environment with easy and scalable screening tools would assist the personalization of medicine and the rationalization of healthcare resources. According to a recent study ^21^, there was a genetic link between covid-19 and AD via the oligoadenylate synthetase 1 (OAS1) gene. This gene is expressed in microglia and is involved in innate immune responses to viral infection, with an increased risk for AD. More specifically, a total of 2547 DNA samples were genotyped for this study, of which 1313 were from sporadic Alzheimer's disease patients and 1234 controls. Through this analysis, the researchers evaluated 4 variants of the OAS1 gene, which resulted in a reduction in its expression. These four single nucleotide polymorphisms (SNPs) are rs4766676 and rs1131454, which have been also linked to AD, and rs6489867 and rs10735079, which are linked to severe symptoms within COVID-19. According to the results of the study, the SNPs within OAS1 that were associated with AD and the ones associated with severe symptoms within COVID-19 showed a linkage disequilibrium (LD). In the same study, some unique genetic expression networks were found in microglial cells that contained genes of interferon response pathways and macrophages treated to mimic Covid-19 effects. More specifically, the gene was found to control the number of pro-inflammatory proteins released by immune system cells. Moreover, it was also found that knockdown of OAS1 expression using small interfering RNA (siRNA) in microglia cells unleashed a “cytokine storm”, which led to exaggerated pro-inflammatory response to tissue damage with an autoimmune state where the immune initiates an attack on itself.

The above study is a first indication of a potential genetic link between AD risk and susceptibility to PACS symptoms centered on the OAS1 gene. However, we are still at the tοp of an iceberg that needs to be explored for better understanding and treatment of diseases such as AD and PACS, as well as for the development of biomarkers to monitor the progression of this interaction. Such studies would enable predictive algorithms for the brain resilience signature and or disease targeting modifiable risk factors such as vascular health maintenance and suggest digital biomarker metrics sensitive to short- and long-term cognitive sequelae that contribute to decreased quality of life and cost to society, especially in diverse populations.

### Postoperative Delirium long-term cognitive outcomes

Delirium remains a commonly encountered condition in older adults post hospitalization, specifically postoperative delirium, occurring 24 to 72 hours after surgery ^22^. The incidence depends on the type of surgery and increases to 15–25% after elective surgery while reaching around 50% of cases in frail or high-risk elderly patients ^23^. Notably, delirium is very common in COVID-19 patients, partly worsened by prolonged mechanical ventilation ^24^. In such situations, within the temporary ICU the formal acute brain dysfunction monitoring is nearly impossible and delirium screening is based on pure clinical observation. Usually it follows a pattern of rapid onset, serious symptom fluctuation during day hours, powerful disruption of the sleep and wake cycle and changes in functional cognition and behavior ^25^. Postoperative period patients who develop delirium had a 10–11% risk of death increase within three months, increased duration of hospitalization and more importantly an increased cognitive impairment risk in the long-term ^26^. It is more common to observe such radical cognition fluctuations long term in frail patients undergoing longer, more-invasive procedures, like cardiac and orthopedic surgery ^27^. Risk factors of developing postoperative delirium could be patient specific predisposing factors, such as frailty or low functional cognition in daily activities, underprivileged education, alcohol abuse history and or any neurological and cognitive impairments that are pre-existing ^28^. It could also be surgery related or medical factors depending on the timing of appearance during hospitalization ^29^. However, given the largely underestimated predementia stages, as explained before, the postoperative delirium incidence could in the future be much larger than conveyed in available literature. Moreover, we hypothesize that given the weighty neurological and neuropsychiatric PACS consequences, which are also present at the younger age groups, the denoting cognitive fluctuations of postoperative delirium would be increasingly more present in a more heterogeneous and potentially younger population.

### Digital Neuro-Signatures of Brain Resilience

With such urgent requirements in mind, two avenues for the investigation of diagnostic and monitoring biomarkers for cognitive function in both PACS and Postoperative delirium can be examined: (1) creating a series of active “digital footprints” for functional cognition and including physical, social and systemic vascular risk, systemic inflammation and metabolic dysfunction determinants as a digital biomarker platform, and/or (2) identifying a unique complex Digital Neuro Signature ^30^ (DNS)™ that exists as a proxy for brain resilience. Such screening tools would be significantly important, since acquiring imaging data of PACS or postoperative delirium patients might be challenging especially for beta-amyloid, tau accumulation, brain glucose use or even MRI-based blood flow ^31^.

To satisfy the first alternative, the study RADAR-AD tried to validate such a platform via a series of passive and active digital biomarkers collected through tablets, smartphones, wearables ^32^ or other sources of the Internet of Things (IoT) and also inferred causality between commercial tools that offer active digital biomarkers with various biological variables. Such platforms might contain different classes of digital biomarkers ranging from diagnostic, prognostic, monitoring, pharmacodynamic, predictive to safety and susceptibility digital biomarkers, depending on their unique structure ^33^. For example, a recent study from UCSF Osher Center for Integrative Medicine, named TermPredict utilized the Oura smart ring for such a platform to rapidly predict coronavirus symptoms before 24 hours ^34^. It should be noted that such platforms are still under validation, focusing on detecting common symptoms for COVID-19 like fatigue and aching muscles, fever, dry cough and shortness of breath, but not brain fog. This is a promising start for a metric that is easy for both patients and researchers to read and interpret, further independent validation is still needed ^35^.

To satisfy the second alternative, a unique DNS monitoring biomarker ^36^ is being evaluated for Remote Data Acquisition (RDA) in the Deep Neuro Study (DNS cohort study), one of four national priority projects supported by the Greek government. This active biomarker type could prove useful in clinical practice as well as treatment options supposing to allow the appraisal of subtle neural correlates of COVID-19 cognitive dysfunction. The DNS monitoring biomarker would serially measure those subtle imbalances, so that changes in metabolic and neurotransmission signatures indicate meaningful changes that predict early neurological damage and the subsequent cognitive decline. Such abilities of novel digital biomarkers to measure off-target effects from molecular signatures such as brain metabolism will increasingly come into play, after a recent draft guidance document from the FDA to provide guidelines to investigators, sponsors and various stakeholders on the use of digital health technologies (DHTs) when acquiring data in clinical investigations remotely ^37^.

The goal of the present open letter is to urgently raise awareness about a pressing issue, possibly increasing the risk of cognitive impairment or AD in the future ^38^, which might prove catastrophic for the healthcare systems in the future. Such awareness is particularly important, especially for at-risk populations in middle and low income regions with missing biomarker studies and with a population that is predisposed due to contributors ^39^ such as obesity, low brain resilience signatures, as well as certain genetic variants. Furthermore, a recent study for Sofie Persson *et al.* ^40^, aimed to explore healthcare costs attributed to dementia for 17 years, underscores the costs that occur one decade before diagnosis. Τimely diagnosis with DNS monitoring biomarkers, would benefit from an economic modeling to estimate the exact costs increase both post screening and six years after diagnosis. Previous work showed that cognitive impairment leading to dementia usually develops 18 years prior to diagnosis ^41^. We postulated that due to the underestimated population of preclinical AD, increased healthcare use could be and should be calibrated several years before the formal dementia diagnosis.

## Data availability

No data are associated with this article.

## Ethics and consent

Ethical approval and consent were not required.
