# Supplementary material for: Making pre-screening for Alzheimer's disease (AD) and Postoperative delirium among post-acute COVID-19 syndrome - (PACS) a national priority: The Deep Neuro Study
Source: Open Res Eur. 2022 Aug 22;2:98. [Version 1] doi: 10.12688/openreseurope.15005.1 (PMC10521085; doi:10.12688/openreseurope.15005.1)
Supplement: Peer review history

# Reviewer report: Reviewer response for version 1

**DOI:** 10.21956/openreseurope.16222.r34776

**Published:** 2023-09-18

**Authors:** Patrizia Vannini

**Affiliations:** 1 Department of Neurology, Brigham and Women's Hospital, Massachusetts General Hospital, Harvard Medical School, Boston, USA

This open letter written by Drs. Tarnanas and Tsolaki is discussing the importance of setting up pre-screening tools for Alzheimer's disease, postoperative delirium among post-acute COVID-19 syndrome and the economical impact this may have on a national level. The rationale for this call is grounded on the fact that there may be 416 million people globally on the AD spectrum of which 315 million is estimated to be in the preclinical stage (cognitively normal but demonstrating evidence of AD biomarkers). The authors suggest the implementation of one, alternatively 2 pre-screening tools - both involving the assessment of different biomarkers assessed through digital health technologies - that would be used to assess risk of future cognitive decline and potential progression to AD in these high risk populations. This is an incredibly important topic and the authors are proposing a very interesting way to address this problem.

 My main issue with the text as written is the lack of detail of what the 2 pre-screening tools consist of, making it hard/or nearly impossible to evaluate their impact and potential use. I would have like to see more details of the assessments for the 'digital footprint' respectively 'digital neuro signature'. What is collected and how is it collected?

 Related to my question above, it is unclear if these assessments would be done on a yearly basis - for instance in conjunction with a general health check-up with an individual's primary care physician or equivalent, or if this is something that is being monitored on a regular basis from home and sent in digitally (again see my question above). If the latter, several question that comes to mind, and which has not been addressed in the text, are: who will collect this data? were will this data be stored? and who will analyze it/disseminate this information back to the individual? This information is important as it impacts the feasibility of the assessments as well as the potential time and effort it will have on the individual.

 I agree with the authors that several challenges exist to assess biomarkers using imaging modalities (e.g. positron emission tomography involving radioactive ligands to assess amyloid, tau and/or glucose metabolism in vivo, and Magnetic Resonance Imaging to assess functional or structural changes) on a bigger scale but I found that there was a lack of discussion about blood biomarkers in this regard. That is, a blood test is a simple and relatively inexpensive approach that can capture a lot of the biological markers the authors are mentioning in their letter and which could be done at yearly health checks. How are the authors approach better in that regard? That is, what would make these digital assessments better and/or more cost-effective?

**Minor issues:**

 Several words needs to be explained before using their abbreviation -e.g. FDG-PET, Ca2+, TGF-B, ICU, RADAR-AD, FDA.

 In some instances in the text COVID-19 is written in small caps.

 Page 4 - third paragraph - the sentence including the words 'day hours' seems to be missing a word and/or the sentence is not grammatically correct.

Where applicable, are recommendations and next steps explained clearly for others to follow? (Please consider whether others in the research community would be able to implement guidelines or recommendations and/or constructively engage in the debate)

Partly

Does the article adequately reference differing views and opinions?

Yes

Are all factual statements correct, and are statements and arguments made adequately supported by citations?

Yes

Is the rationale for the Open Letter provided in sufficient detail? (Please consider whether existing challenges in the field are outlined clearly and whether the purpose of the letter is explained)

Yes

Is the Open Letter written in accessible language? (Please consider whether all subject-specific terms, concepts and abbreviations are explained)

Partly

Reviewer Expertise:

Aging and Alzheimer's disease, biomarker assessment and pre-clinical markers,

I confirm that I have read this submission and believe that I have an appropriate level of expertise to confirm that it is of an acceptable scientific standard, however I have significant reservations, as outlined above.

# Reviewer report: Reviewer response for version 1

**DOI:** 10.21956/openreseurope.16222.r34779

**Published:** 2023-09-18

**Authors:** Miguel Vaz

**Affiliations:** 1 University of Beira Interior, Covilhã, Portugal

The open letter written by Tarnanas *et al.* deals with a very current and important topic. The authors address the cognitive deficit associated with post-acute COVID-19 syndrome and the mechanism that appears to be behind it. They suggest the use of selected digital biomarkers to early detect cognitive impairment/Alzheimer’s Disease.

 It is a well-structured article but, in my opinion, it would benefit from a more detailed explanation of how these digital tools work and a critical view of the ethics of early diagnosis. This screening could lead to overdiagnosis, especially when none of the biomarkers can fully predict the natural course of dementia and, furthermore, its detection remains expensive. I recommend reading the following article for support: “Preclinical Alzheimer’s dementia: a useful concept or another dead end?” (DOI: 10.1007/s10433-022-00735-w ^1^).

 Additionally, all the arguments are adequately supported by citations.

 The authors use accessible language. However, I must highlight some minor issues:

- Some abbreviations that are not well identified (e.g: COVID-19 is not explained the first time it appears in the abstract, “mo” and “ICU” are not explained).
- Covid-19, COVID-19 and Covid-19 – These 3 forms appear throughout the text, I suggest harmonisation of the text.
- In the subchapter “ *PACS long-term cognitive outcomes*”, when the authors describe the “ *…leaky ryanodine receptor 2 (RyR2) even following mild illness and across all ages10*”, reference 10 does not seem to be well formatted.
- I recommend placing references at the end of the sentence and not in the middle (e.g. “ *A recent comprehensive investigation ^14^ in the context of brain…*”).

Where applicable, are recommendations and next steps explained clearly for others to follow? (Please consider whether others in the research community would be able to implement guidelines or recommendations and/or constructively engage in the debate)

Yes

Does the article adequately reference differing views and opinions?

Partly

Are all factual statements correct, and are statements and arguments made adequately supported by citations?

Yes

Is the rationale for the Open Letter provided in sufficient detail? (Please consider whether existing challenges in the field are outlined clearly and whether the purpose of the letter is explained)

Yes

Is the Open Letter written in accessible language? (Please consider whether all subject-specific terms, concepts and abbreviations are explained)

Partly

Reviewer Expertise:

Alzheimer's Disease, Neurodegenerative disease, pharmacology

I confirm that I have read this submission and believe that I have an appropriate level of expertise to confirm that it is of an acceptable scientific standard.

## References

[1] : Preclinical Alzheimer's dementia: a useful concept or another dead end?. *Eur J Ageing* .2022;19(4) : 10.1007/s10433-022-00735-w 997-1004 10.1007/s10433-022-00735-w 36506684PMC9729660

# Reviewer report: Reviewer response for version 1

**DOI:** 10.21956/openreseurope.16222.r34262

**Published:** 2023-08-30

**Authors:** Jolanta Dorszewska, Mikołaj Hurła, Natalia Banaszek

**Affiliations:** 1 Laboratory of Neurobiology, Department of Neurology, Poznan University, Poznan, Poland; 2 Poznan University of Medical Sciences, Poznan, Poland

The open letter "Making pre-screening for Alzheimer's disease (AD) and Postoperative delirium among post-acute COVID-19 syndrome - (PACS) a national priority: The Deep Neuro Study" is part of the currently popular research topic and important for practice clinically. The high level of preparation of the article deserves to be emphasized. I have a few comments on the authors of this article. I am asking the authors to consider improving the articles as indicated, which will make it easier for readers to understand the subject matter.

1. The rationale for the letter is well structured and thoroughly explains why there is a need for such a pursue in research regarding pre-screening for AD and Postoperative delirium among PACS patients. The author explains the mechanism leading to potential diseases, shows the size of the potentially susceptible groups, and concludes up-to-date efforts that haven't met the said goal.
2. The author should consider a paragraph explaining more thoroughly the currently available equipment for digital data collection as well as the limitations of said technologies.
3. Conclusion. Is it possible to think about an early diagnosis of dementia if the currently used drugs only slow down the progression of the disease and do not have a causal effect? Ethical problem. Consider a different take on the summary.
4. All of the given facts and arguments seem to correspond with the given citations.
5. The language of this open letter is clearly accessible and easily understandable

Where applicable, are recommendations and next steps explained clearly for others to follow? (Please consider whether others in the research community would be able to implement guidelines or recommendations and/or constructively engage in the debate)

Partly

Does the article adequately reference differing views and opinions?

Yes

Are all factual statements correct, and are statements and arguments made adequately supported by citations?

Yes

Is the rationale for the Open Letter provided in sufficient detail? (Please consider whether existing challenges in the field are outlined clearly and whether the purpose of the letter is explained)

Yes

Is the Open Letter written in accessible language? (Please consider whether all subject-specific terms, concepts and abbreviations are explained)

Yes

Reviewer Expertise:

Neurodegenerative diseases, including Alzheimer's and Parkinson's. Neurobiology and neurogenetics.

We confirm that we have read this submission and believe that we have an appropriate level of expertise to confirm that it is of an acceptable scientific standard.
